# Chronological lifespan extension and nucleotide salvage inhibition in yeast by isonicotinamide supplementation

**DOI:** 10.1016/j.jbc.2026.111158

**Published:** 2026-01-13

**Authors:** Agata I. Kalita, Christopher T. Letai, Elisa Enriquez-Hesles, Lindsey N. Power, Swarup Mishra, Shekhar Saha, Manikarna Dinda, Dezhen Wang, Pankaj K. Singh, Jeffrey S. Smith

**Affiliations:** 1 Department of Biochemistry and Molecular Genetics, University of Virginia School of Medicine, Charlottesville, Virginia, USA; 2 Faculty of Biochemistry, Biophysics and Biotechnology, Jagiellonian University, Cracow, Poland; 3 Department of Biology, University of Virginia, Charlottesville, Virginia, USA; 4 Department of Microbiology, Immunology & Cancer Biology, University of Virginia School of Medicine, Charlottesville, Virginia, USA; 5 Eppley Institute for Cancer Research, University of Nebraska Medical Center, Nebraska, USA; 6 Department of Oncology Science, Oklahoma University College of Medicine, Oklahoma City, Oklahoma, USA

**Keywords:** chronological aging, isonicotinamide, NAD^+^, nucleotides, nucleotidase, yeast

## Abstract

Isonicotinamide (INAM) is an isomer of the NAD^+^ precursor nicotinamide (NAM) that stimulates the enzymatic activity of Sir2, an NAD^+^-dependent histone deacetylase from the budding yeast, *Saccharomyces cerevisiae*. Supplementing INAM into growth media promotes the replicative lifespan of this single cell organism by maintaining intracellular NAD^+^ homeostasis. INAM also extends yeast chronological lifespan, but the underlying mechanisms remain largely uncharacterized. To identify cellular pathways potentially impacted by INAM, in this study we perform a chemical genomics screen of the yeast knockout collection for mutants sensitized to growth inhibition by INAM. Significant Gene Ontology terms for candidate genes include transcription elongation factors, metabolic pathways converging on one-carbon metabolism, and *de novo* purine biosynthesis, collectively suggesting that INAM perturbs nucleotide metabolism. Indeed, INAM causes dose-dependent depletion of intracellular cytidine, uridine, and guanosine, ribonucleosides derived from the breakdown of nucleotide monophosphates (NMPs) *via* nucleotidases (Phm8, Sdt1, and Isn1) or the alkaline phosphatase Pho8. We also find that INAM directly inhibits recombinant nucleotidase activity using cytidine or nicotinamide mononucleotide as substrates and inhibits alkaline phosphatase activity quantitated from whole cell extracts. Finally, we found that Phm8 and Pho8 are specifically required for INAM-induced chronological lifespan extension, implicating them as likely functional targets *in vivo*. Taken together, the findings suggest a model whereby partial impairment of nucleotide and/or NAD^+^ salvage pathways by INAM can trigger a hormetic stress response that supports enhanced quiescence during chronological aging.

Aging is a multifaceted process leading to decreased function over time, and ultimately, death of the cell or organism. Despite extraordinary variance in organism lifespans, many of the proteins and pathways involved in aging are evolutionarily conserved (1, 2), making research in short-lived, experimentally feasible model organisms applicable to human aging. For example, budding yeast (*Saccharomyces cerevisiae*) has played a major role in the identification and characterization of several key longevity or health span factors, including the NAD^+^-dependent histone deacetylase Sir2 (reviewed in (3)), the founding member of the conserved sirtuin protein family (4). Sir2 is critical for sustaining the replicative potential of yeast mother cells, also known as their replicative lifespan (RLS), primarily through stabilizing the repetitive rDNA locus (5). In contrast, shown in further work in yeast, Sir2 limits the long-term survival of nonproliferating cells in stationary phase, also known as chronological lifespan (CLS), due to specific Lys-514 deacetylation (inactivation) of the rate-limiting gluconeogenesis protein Pck1 (6). Gluconeogenesis improves ethanol and acetate utilization and elevates glycogen/trehalose storage, thus enhancing stress resistance and CLS (7).

Sirtuin-mediated lysine deacetylation produces nicotinamide (NAM) as a byproduct, which can feed-back and inhibit the reaction if not recycled by the NAD^+^ salvage pathway (8, 9, 10). NAM supplementation at high concentrations therefore phenocopies the effect of deleting the *SIR2* gene, reducing RLS and extending CLS (9, 11). Biochemically, NAM inhibits deacetylation activity by regenerating acetyllysine and NAD^+^ through a base exchange reaction with the peptidyl-imidate intermediate (12). Isonicotinamide (INAM), a nonreactive isostere of NAM (Fig. 1*A*), can relieve the Sir2 NAM inhibition and stimulate deacetylation activity by blocking the NAM binding pocket (13). Supplementing INAM into media therefore enhances heterochromatic gene silencing and RLS in a Sir2-dependent manner (13, 14). However, rather than shortening CLS, as would be predicted for enhanced Sir2 activity, INAM extends CLS independently of Sir2 (15), suggesting the involvement of potentially novel CLS regulatory mechanisms.

**Figure 1:**
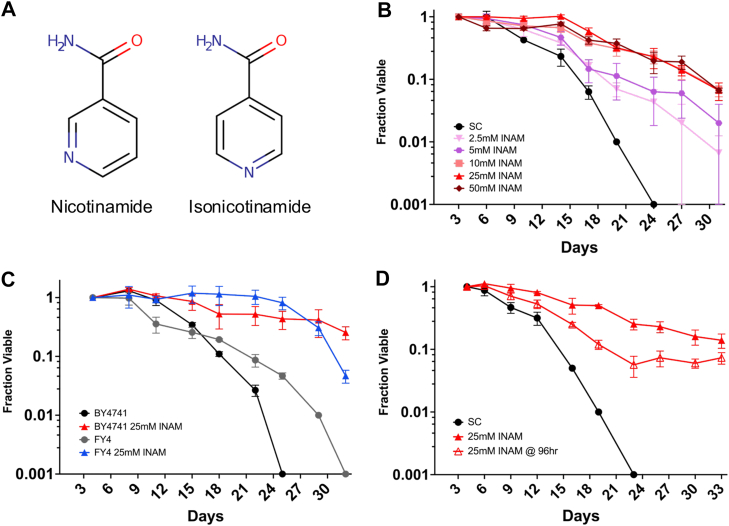
**INAM extends chronological lifespan**. *A*, chemical structures of nicotinamide and isonicotinamide. *B*, CLS assays with increasing dosage of INAM added to BY4741 at the time of inoculation. *C*, CLS assay comparing the effect of 25 mM INAM on the auxotrophic strain BY4741 and prototrophic strain FY4, added at inoculation. *D*, CLS assay of BY4741 when 25 mM INAM is added 96 h after inoculation. Error bars indicate standard deviations (n = 3 biological replicates). OASIS2 statistics for CLS assays are provided in Table S5. CLS, chronological lifespan; INAM, isonicotinamide.

Although INAM stimulates the histone deacetylase (HDAC) activity of recombinant Sir2 *in vitro* (13), it also promotes Sir2-dependent transcriptional silencing and RLS *in vivo* by maintaining intracellular NAD^+^ homeostasis when NAD^+^ precursors (NAM or nicotinic acid) are limiting (14). INAM is not a direct NAD^+^ precursor, so its effects on NAD^+^ homeostasis likely involve the modulation of one or more steps of the *de novo* synthesis or salvage pathways. Consistent with this idea, NAM supplementation during chronological aging maintains the expression of several genes in these pathways when cells enter stationary phase, including the nicotinic acid/nicotinamide mononucleotide adenylyltransferase *NMA1* (14). INAM also upregulates the expression of genes involved in cell wall biosynthesis and remodeling, likely related to concurrent protection from acetic acid toxicity (15). However, the biochemical targets underlying these longevity-associated phenotypes remain unknown. Identifying such targets could provide important CLS mechanistic insights.

To identify possible INAM-targeted cellular processes, in this study, we utilize an unbiased chemical synthetic lethality screening approach with the yeast knockout (YKO) strain collection. We hypothesized that concentrations of INAM that extend CLS may moderately inhibit specific cellular processes, which becomes growth inhibitory when combined with deletion of a gene from an interacting pathway or process. We find that deletion mutants growth-inhibited by sublethal INAM concentrations cluster into several functional Gene Ontology (GO) terms, including transcription elongation factors. A similar synthetic lethal pattern was previously observed with mycophenolic acid (MPA), a compound that inhibits IMP dehydrogenase and depletes the guanine nucleotide pool (16, 17). However, rather than specific reduction of guanine nucleotide biosynthesis, we find that INAM acutely impairs the salvage of ribonucleoside monophosphates CMP, GMP, UMP, and the NAD^+^ precursor, nicotinamide mononucleotide (NMN) to their respective nucleosides by inhibiting a set of nucleotidases and the alkaline phosphatase, Pho8. Ultimately, overall nucleotide triphosphate pools become limited as cells enter stationary phase. We propose a model whereby moderately limiting the nucleotide pool through inhibition of nucleotidase and alkaline phosphatase activity by INAM induces a beneficial hormesis response that improves CLS.

## Results

### INAM-induced CLS extension is titratable and independent of auxotrophies

To begin characterizing the mechanism of INAM-induced CLS extension, we tested for dosage effects on the YKO parental strain BY4741. Previous *in vivo* experiments with INAM were performed at 25 mM, a concentration that promotes Sir2-dependent transcriptional silencing (13). In Figure 1*B*, concentrations ranging from 2.5 to 50 mM were supplemented into CLS cultures at the time of inoculation. Lifespan plateaued at 10 to 25 mM, while 50 mM still extended CLS despite causing reduced viability at day 3. To rule out contributions of the auxotrophic mutations in BY4741 to CLS extension, which have been shown to significantly impact longevity in the yeast system (18), we showed that 25 mM INAM extended CLS of the prototrophic progenitor strain FY4 ((19); Fig. 1*C*). In addition, 25 mM extended CLS of BY4741 when added to cultures at day 4, after the cells entered stationary phase (Fig. 1*D*). The effect was not as strong as supplementing at the time of inoculation, suggesting the underlying mechanisms impact both proliferating and quiescent cells.

### INAM extends CLS independently of sirtuins

**Figure 2:**
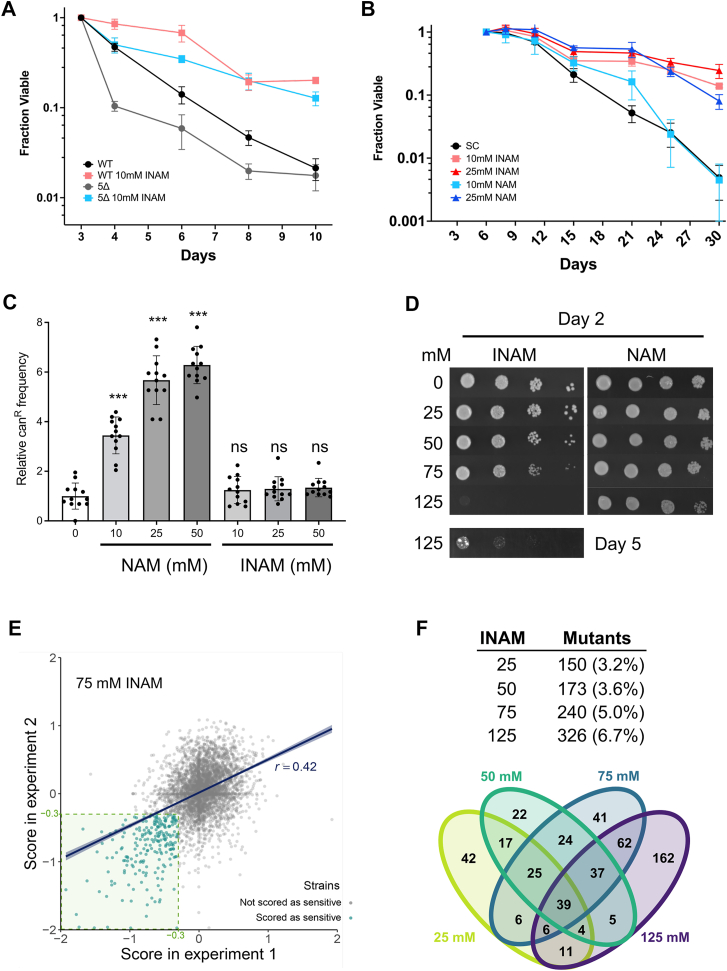
**INAM effects on CLS are independent of sirtuins**. *A*, CLS assay demonstrating that 10 mM INAM extends CLS of a quintuple sirtuin (*sir2Δ*, *hst1Δ*, *hst2Δ*, *hst3Δ*, and *hst4Δ*) mutant. *B*, CLS assay comparing the effects of INAM and NAM supplemented to BY4741 at 10 or 25 mM. OASIS2 statistics for CLS assays are provided in Table S5. *C*, relative mutation frequency caused by overnight growth on NAM or INAM at indicated concentrations. The average frequency for the control without supplements was set at 1.0. Error bars indicate standard deviation (n = 12), and *asterisks* indicate *p* values <0.001 when compared to the control (one-way ANOVA test). *D*, growth of BY4741 with increasing concentrations of INAM or NAM on SC media. 10-fold serial dilutions of cells. The 125 mM INAM plate was also incubated to day 5. CLS, chronological lifespan; INAM, isonicotinamide; NAM, nicotinamide; SC, synthetic complete. *E*, scatter plot of scores from each deletion strain obtained in experiment 1 (x-axis) and experiment 2 (y-axis). Strains with SGAtools fitness scores lower than −0.3 in both replicates were considered sensitive to a given INAM concentration (representative graph for 75 mM INAM). *F*, number of mutants identified for each INAM concentration, including percent coverage of all genes, and a Venn diagram indicating the overlaps.

We previously found that INAM supplementation suppressed the NAD^+^ depletion that normally occurs as yeast cells enter stationary phase (14, 20). Despite the association of elevated NAD^+^ with increased *SIR2* function, INAM-induced CLS extension did not require *SIR2* (15). Significant functional redundancy exists between Sir2 and its paralog Hst1 (21, 22, 23), so to rule out contributions from Hst1 or the other Sir2 homologs, we tested whether INAM extended CLS of a mutant lacking all five sirtuin genes, *SIR2*, *HST1*, *HST2*, *HST3*, and *HST4* (4). CLS of the quintuple sirtuin mutant was reduced compared to the control, but 10 mM INAM still significantly extended lifespan (Fig. 2*A*), suggesting that while improved NAD^+^ homeostasis may play a role in the CLS benefit, sirtuins are not required.

The lack of sirtuin involvement suggested that despite being a sirtuin inhibitor, NAM could also potentially extend CLS due to its chemical similarity with INAM. We previously reported that 5 mM NAM did not extend CLS when added to cultures at the time of inoculation (24), but an independent study found that NAM extended CLS when added during the diauxic shift (11). NAM at 5 mM previously inhibited Sir2, Hst1, Hst2, and Hst4, while 25 mM NAM was required to inhibit Hst3 and ensure the elimination of all sirtuin function (9, 25). In Figure 2*B*, we observed that 25 mM NAM significantly extended CLS of BY4741, but 10 mM NAM had little effect compared to 10 mM INAM, indicating that INAM is the more potent isomer by this measure. NAM also has mutagenic potential at these concentrations due to the inhibition of Hst3 and Hst4, which function as H3K56 deacetylases in the maintenance of genome stability (26, 27). Indeed, NAM supplementation significantly increased mutation frequency of the endogenous *CAN1* reporter gene (canavanine resistant colonies) as previously reported (27), while INAM at the same concentrations had no effect (Fig. 2*C*). INAM is therefore a nonmutagenic CLS booster in comparison to NAM. At concentrations above 50 mM, however, INAM inhibited cell growth while NAM did not (Fig. 2*D*). This led to the hypothesis that lifespan extension at lower INAM concentrations could be due to mild perturbation of one or more activities/pathways that function in cell proliferation.

### A chemical genomic screen for INAM-sensitive deletion strains

To identify genes that buffer against INAM-impaired cellular processes, the *MAT*a haploid YKO strain collection from Euroscarf (4839 mutants) was pinned onto synthetic complete (SC) rectangular agar plates containing 0, 25, 50, 75, or 125 mM INAM (Fig. S1*A*). After 2 days incubation (3 days for 125 mM), the plates were imaged and growth of each mutant colony on the INAM-containing plates was quantitated relative to SC control plates using SGAtools (28). Examples of colony growth on SC and SC + 50 mM INAM for plate #2 are shown in Figure S1*B*. A zoomed-in example from plate #7 showing INAM sensitivity of the *tho2Δ* mutant is shown in Figure S1*C* (center colony, yellow arrow). Growth of all strains was strongly reduced at 125 mM.

The full screen was independently performed twice with 4839 mutants, resulting in moderate but significant correlation in SGAtools-generated fitness scores, as shown for 75 mM INAM in Figure 2*E* (r = 0.42; *p* < 0.00001). We conservatively focused on mutants with a fitness score lower than −0.3 (difference visible by eye; (28)) in both replicates (Tables S1 and S2, Fig. 2*E* inset). The number and percentage of mutants meeting these criteria for each INAM concentration is indicated in Figure 2*F*. Fewer mutants met the −0.3 cutoff at lower concentrations, which was expected as the degree of pathway disruption should be weaker (Fig. 2*F*). Only 39 mutants were identified from all four concentrations, though there were greater overlaps for any two concentrations (Fig. 2*F*). Approximately 50% of the 326 mutants chosen as sensitive to 125 mM INAM at day 3 did not overlap with mutants from the other concentrations (Fig. 2*F*), suggesting a high number of false positives for this highest concentration. We therefore focused GO enrichment analysis on a collection of 341 genes identified from at least one of the 25, 50-, or 75-mM conditions (Table S1). Several “Biological Process” GO terms with significant *p* values were related to transcriptional regulation, autophagy, vacuolar transport, inositol phosphate biosynthesis, and chromatin remodeling (Table S3). “Cellular Component” GO terms covered the INO80 and SWR1 chromatin remodeling complexes, transcriptional elongation factors, endosomes and the endosomal sorting complex required for transport (ESCRT) complex (Table S3). INAM sensitivity was confirmed for 57 of 61 retested deletion mutants (Figs. S2 and S3), indicating the cutoff score of −0.3 was highly stringent. Based on predictions from the enriched GO terms and literature searches, 22 additional mutants related to the GO terms were directly tested and confirmed as INAM-sensitive (Fig. S4).

### Disruption of serine, glycine, threonine, or cysteine metabolism confers sensitivity to INAM

**Figure 3:**
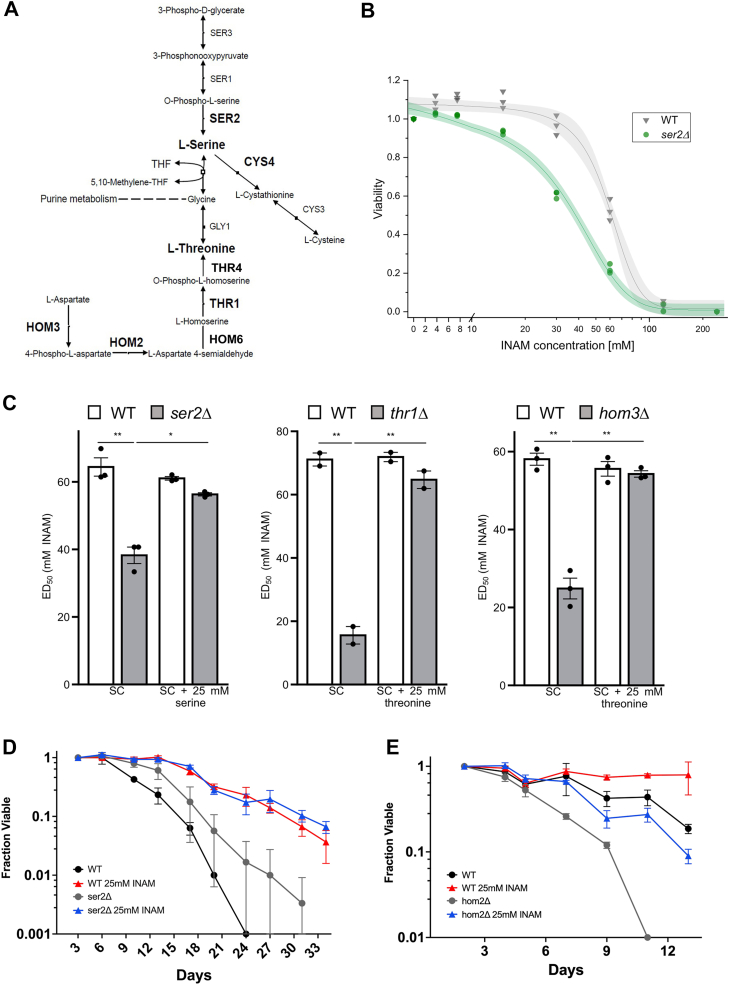
**Serine and threonine biosynthesis mutants are sensitive to INAM**. *A*, a diagram illustrating the synthesis pathways of serine, aspartate, threonine, and cysteine, as well as the integration into one-carbon metabolism and purine metabolism. Deleted genes conferring INAM sensitivity are in *bold*. *B*, representative quantitative dose-response curve illustrating the relative viability of BY4741 (WT) and the *ser2Δ* mutant at a range of INAM concentrations. *Dots* and *inverted triangles* represent values obtained from three biological replicates with sigmoid curves fitted to the data using a dose response function and the Levenberg–Marquardt iteration algorithm. *Shaded* areas around the curve are 95% confidence bands. *C*, mean INAM effective dose (ED_50_) values for BY4741 (WT), *ser2Δ*, *thr1Δ*, *and hom3Δ* strains, indicating concentration from the growth curves in *panel B* where the relative growth (viability) equals 0.5. *p* values were calculated by an unpaired *t* test. ∗*p* < 0.05, ∗∗*p* < 0.005. Three biological replicates were included, except for *thr1Δ*, which had two replicates. *D*, CLS assay showing moderate lifespan extension of *ser2Δ* mutant, and full extension by 25 mM INAM. *E*, CLS assay showing shortened lifespan of a *hom2Δ* mutant that is partially restored by 25 mM INAM. OASIS 2 statistics for CLS assays are provided in Table S5. N = 3 biological replicates. CLS, chronological lifespan; INAM, isonicotinamide.

Intriguing metabolism-related GO terms enriched in the 25, 50, or 75 mM INAM-sensitive mutant lists were *de novo* IMP biosynthesis (*ADE1*, *ADE4*, *ADE6*, and *ADE5*,*7*), threonine metabolic process (*HOM2*, *HOM3*, *THR4*, and *GLY1*), and serine family amino acid biosynthetic process (*CYS4*, *GLY1*, *HOM2*, *HOM3*, *SER1*, and *SER2*) (Table S3). Figure 3*A* depicts connections between these enzymes, with the points of convergence at serine and glycine, amino acids that fuel one-carbon (1C) metabolism for utilization in purine and thymidylate (dTMP) synthesis, methionine/SAM regeneration, amino acid synthesis, and redox maintenance (29). Serine metabolism was previously linked to CLS regulation, with serine supplementation extending CLS (30, 31), and *SER1* identified as a major quantitative trait locus (QTL) for genetic CLS variation among strain backgrounds (32). The serine/glycine/threonine pathways were also reported as a metabolic axis for longevity regulation based on multi-omics analyses in mice (33). Although INAM clearly interacts with this metabolic axis, deletion mutants for the core one-carbon metabolism genes were not isolated from the sensitivity screen, except for *ade3Δ*, which is also critical for the *de novo* purine synthesis pathway.

Numerous metabolic enzymes “moonlight” with biochemical functions separate from their annotated catalytic activity (34). We therefore wanted to confirm that the serine or threonine metabolism mutants were INAM-sensitive due to a serine or threonine deficit, respectively. To quantify the growth effects, an effective dose of INAM that reduced growth by 50% (ED_50_) was calculated from small liquid cultures in 96-well plates, as shown for *ser2Δ* in Figure 3*B*. Supplementing 25 mM serine indeed restored normal growth to the *ser2Δ* mutant and, similarly, 25 mM threonine restored growth to the more severe *thr1Δ* and *hom3Δ* mutants (Fig. 3*C*). Even though these mutants were sensitive to high concentrations of INAM, their CLS was still extended by 25 mM INAM (Fig. 3, *D* and *E*; data shown for *ser2Δ* and *hom2Δ*). Note that the *hom2Δ* mutant was short-lived compared to WT, indicating a role for this pathway in supporting CLS. Poor growth of the *thr1Δ* mutant was incompatible with CLS assays. Taken together, these results suggest that INAM does not function by directly modifying serine, threonine, or glycine metabolic pathways. Rather, defects in availability of serine, glycine, or threonine *via* their biosynthesis or import impair growth when INAM perturbs a functionally linked cellular process.

### Mutants in SWR1 complex subunits and other transcriptional elongation factors are sensitive to INAM

**Figure 4:**
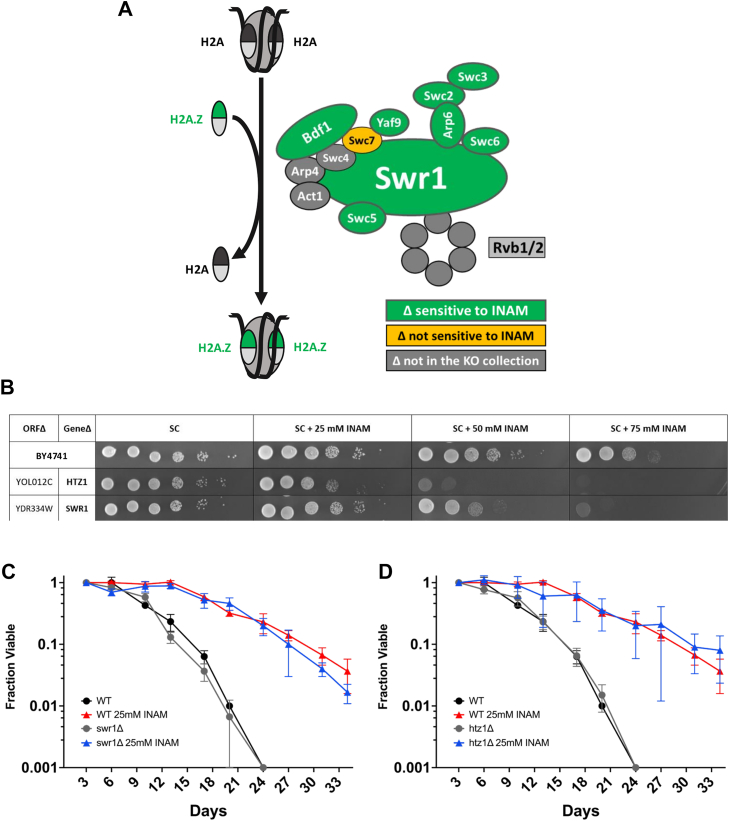
**Strains defective for the H2A.Z deposition by SWR1 are sensitive to INAM**. *A*, illustration depicting the SWR1 activity of H2A/H2A.Z (Htz1) exchange, as well as subunit composition and SWR1 complex. *Green* indicates subunit deletions that were sensitive to INAM and *yellow* indicates not sensitive. *B*, spot test growth assay showing confirmation of INAM sensitivity of *htz1Δ* and *swr1Δ* strains from the YKO collection. The WT strain is BY4741. Samples were not next to each other on the agar plates, so the spliced images are indicated a *black horizontal line*. *C*, CLS assay of BY4741 and *swr1Δ* mutant strains supplemented with 25 mM INAM. *D*, CLS assay of BY4741 and *htz1Δ* strains supplemented with 25 mM INAM. Controls were supplemented with equal volume of sterile water instead of INAM stock solution. OASIS 2 statistics for CLS assays are provided in Table S5. N = 3 biological replicates. CLS, chronological lifespan; INAM, isonicotinamide; YKO, yeast knockout.

Prominent nonmetabolic GO terms included those related to transcriptional regulation and chromatin remodeling, especially at the level of transcriptional elongation (Table S3). Among these were multiple subunits of the SWR1 complex, a conserved ATP-dependent chromatin remodeler that exchanges H2A core histones in nucleosome octamers for the H2A.Z variant encoded by *HTZ1* (Fig. 4*A*; (35, 36)). Exchange of H2A with H2A.Z (Htz1) by SWR1 impacts multiple steps of gene expression, including facilitating progression of polymerase through nucleosomes during transcription elongation (37). Mutants for five SWR1 subunits (*bdf1Δ*, *vps72Δ*, *swc5Δ*, *swr1Δ*, and *yaf9Δ*) were identified from the screen at 25, 50, or 75 mM. Deletions for three additional annotated SWR1 mutants (*arp6Δ*, *swc3Δ*, *swc7Δ*) were tested separately. The *swc7Δ* mutant was the only SWR1 candidate we tested that was not INAM-sensitive (Fig. 4*A*), most likely because the Swc7 subunit is not required for H2A.Z binding or histone replacement activity (38). The *htz1Δ* strain was also isolated from the screen and confirmed as sensitive at 25 mM (Fig. 4*B*). Several other factors associated with elongation were identified from the INAM sensitivity screen, including subunits of the THO complex, the elongator complex, and the C-terminal domain kinase complex (CTDK-I), suggesting that INAM impairs one or more cellular processes that support transcriptional elongation.

SWR1 subunit mutations were previously shown to extend CLS of a prototrophic strain in nitrogen-rich media and to prevent the CLS extension induced by nitrogen-poor media, a form of caloric restriction (39). We therefore tested if deleting *SWR1* or *HTZ1* would prevent CLS extension induced by INAM. Surprisingly, neither mutant affected CLS with or without 25 mM INAM supplementation (Fig. 4, *C* and *D*), suggesting any transcriptional or chromatin defects associated with SWR1 were not significantly contributing to longevity under the conditions of this study. Instead, synthetic lethality of INAM with elongation factor mutants, as well as *de novo* IMP biosynthesis pathway (*ade*) mutants, pointed toward possible alterations in nucleotide metabolism upon INAM supplementation that could impact CLS.

### INAM acts synergistically with MPA

**Figure 5:**
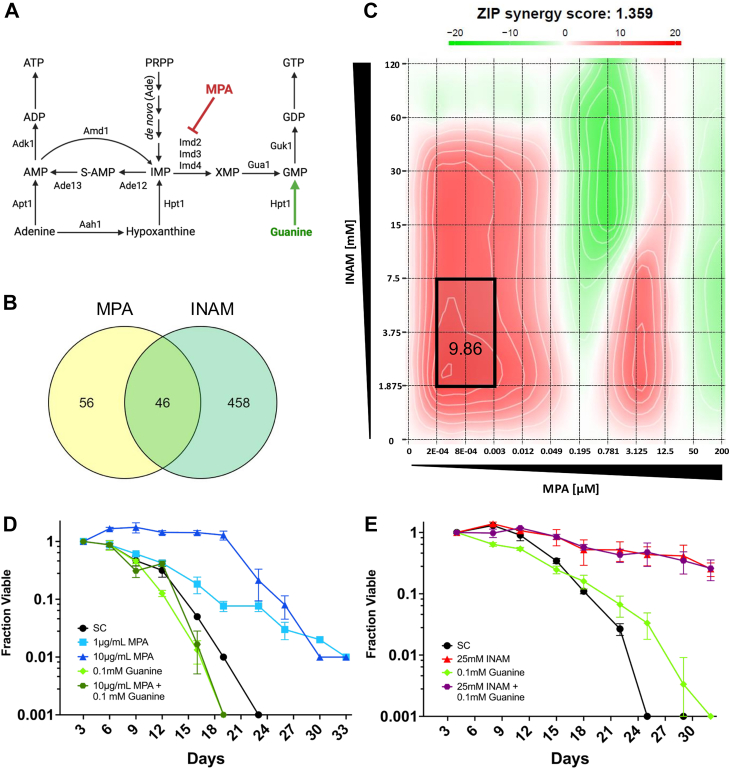
**Mycophenolic acid and INAM operate through related but distinct mechanisms**. *A*, illustration of MPA inhibiting IMP dehydrogenase (Imd2) to reduce guanylic nucleotide synthesis. Guanine salvage *via* Hpt1 (*green*) compensates to support GMP, GDP, and GTP homeostasis. *B*, Venn diagram depicting significant overlap between INAM- and MPA-sensitive deletion strains identified in a chemogenomic screen and confirmed by spot assays. The hypergeometric test *p* value for this overlap (n = 4839 total mutants in the KO collection) is 9.5 x 10^-20^. *C*, synergy map depicting interaction between INAM and MPA in reducing cell growth. Graph is representative of three independent experiments. *D*, CLS assay with BY4741 showing CLS extension by MPA supplementation (1 or 10 μg/ml), and suppression of the effect by 0.1 mM guanine. *E*, CLS assay with BY4741 showing INAM extension of CLS is that is not suppressed by guanine. OASIS 2 statistics for CLS assays are provided in Table S5. N = 3 biological replicates. CLS, chronological lifespan; INAM, isonicotinamide; MPA, mycophenolic acid.

Yeast mutants defective for transcriptional elongation are sensitive to MPA and 6-azauracil (6AU) (40). MPA is a noncompetitive inhibitor of inosine 5′-monophosphate (IMP) dehydrogenase (IMPDH) (16), the enzyme that converts IMP to XMP during *de novo* synthesis of guanine nucleotides (Fig. 5*A*). 6AU is converted into 6-azaUMP, which also inhibits IMPDH (Imd2), as well as orotic acid decarboxylase (Ura3), the last step of UMP biosynthesis (41). The resulting guanine ribonucleotide pool depletion, and UTP depletion in the case of 6AU, impairs transcription elongation and inhibits growth when combined with mutations that slow elongation (42, 43). Comparing the INAM-sensitive mutants with previously identified MPA-sensitive mutants (40), there was significant overlap between the datasets (*p* = 1.41 x 10^-38^; Fisher’s exact test), with 45.1% (46/102) of MPA-sensitive mutants also identified as INAM-sensitive (Fig. 5*B*). Among the overlaps were *htz1Δ* and multiple transcriptional elongation factors, suggesting that INAM is modifying ribonucleotide pools like MPA. Dose response growth curves of BY4741 with combinations of INAM and MPA in liquid cultures revealed strong synergistic growth inhibition (peak ZIP score of 9.86) at relatively low concentrations of each compound that had no effects individually (Fig. 5*C*), implying the two compounds were likely impacting growth through different mechanisms.

We next considered the possibility that INAM was extending *S*. *cerevisiae* CLS by specifically perturbing guanine nucleotide pools through a mechanism different from MPA. As shown in Figure 5*D*, MPA extended CLS at 1 μM, and even more effectively at 10 μM, consistent with its known extension of RLS (44) and *Saccharomyces pombe* CLS (45). As predicted, CLS extension by MPA was fully reversed by supplementing with 0.1 mM guanine (Fig. 5*D*), which bypasses IMP dehydrogenase inhibition by conversion to GMP *via* the salvage enzyme hypoxanthine-guanine phosphoribosyltransferase (Hpt1; Fig. 5*A*). In contrast, CLS extension induced by 25 mM INAM was not reversed by guanine (Fig. 5*E*), implying that IMP dehydrogenase was not directly inhibited by INAM. Ura3 also cannot be a relevant INAM target because BY4741 and the YKO collection strains are all *ura3Δ0*.

### INAM perturbs nucleotide metabolism

**Figure 6:**
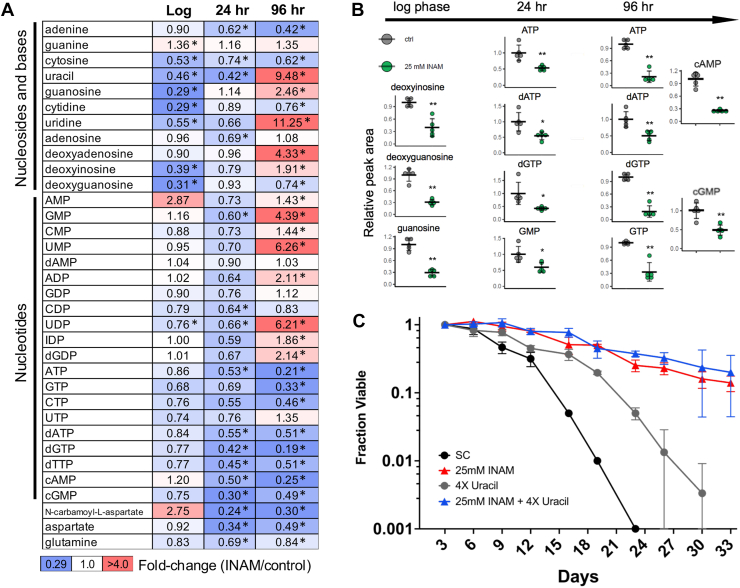
**Alterations of intracellular nucleotide metabolite levels by 25 mM INAM**. *A*, summary of quantitative mass spectrometry analysis for a *panel* of purine and pyrimidine nucleotides, nucleosides, bases, and precursors. *Block* shading indicates fold-change relative to the control without INAM (*red*-up and *blue*-down). *Asterisks* indicate significant changes with *p* values <0.05 as determined by one-way ANOVA, with three biological replicates. *B*, individual examples of significantly reduced purine nucleosides or nucleotides reduced by INAM at each time point. ∗*p* < 0.05, ∗∗*p* < 0.01. *C*, CLS assay showing partial lifespan extension when BY4741 (uracil auxotroph) is supplemented with 4x (0.8 mM) the normal concentration of uracil (0.2 mM) in SC medium. INAM (25 mM) fully extends CLS in combination with 4x uracil. OASIS 2 statistics for the CLS assays are provided in Table S5. CLS, chronological lifespan; INAM, isonicotinamide; SC, synthetic complete.

The combination of INAM sensitivity for mutants defective in transcriptional elongation, *de novo* purine biosynthesis (*ADE* genes), or serine/threonine/glycine biosynthesis pathways, as well as synergistic growth inhibition with MPA suggested a broader effect on nucleotide metabolism. To test this idea, we utilized mass spectrometry to quantify a panel of nucleotides and precursors when BY4741 was grown in the presence of 25 mM INAM (Table S6). Extracts were isolated from treated and untreated cells grown to log phase (6 h), late diauxic shift (24 h), or stationary phase (96 h). Several nucleosides and bases were significantly reduced by INAM in log phase cells (Fig. 6*A*). Although INAM did not significantly affect nucleotide levels in log phase cells, there was still a trend toward reduction of NTPs and dNTPs that became more significant at the 24 h and 96 h time points (Fig. 6, *A* and *B*). These changes were accompanied by reduced aspartate and glutamine, amino acids that contribute atoms to *de novo* biosynthesis of the purine and pyrimidine rings. UTP was the lone NTP/dNTP exception that was not significantly reduced by the 96 h time point in INAM-treated cells (Fig. 6*A*). Moreover, uracil, uridine, UMP, and UDP were all strongly upregulated at 96 h. These changes could be related to the *ura3Δ0* mutation in BY4741 that blocks *de novo* UMP synthesis, forcing the cells to salvage uracil from the media. We therefore tested whether supplementing extra uracil into the growth media would extend CLS and/or modify the lifespan extension induced by INAM. Adding uracil at 4x the normal concentration in SC media did significantly extend CLS but had little impact on lifespan extension induced by 25 mM INAM (Fig. 6*C*).

INAM also reduced cAMP and cGMP levels at the 24 h and 96 h time points, likely reflecting the reduction in ATP and GTP. We were curious if an accompanying reduction of cAMP-dependent protein kinase activity would induce a stress-induced transcriptional program downstream of Rim15 mediated by the stress-induced transcription factors Msn2 and Msn4. This pathway mediates the extension of RLS and CLS induced by CR (46, 47). A lacZ reporter gene under control of a stress response element (STRE) was therefore tested for possible activation by INAM in WT, *rim15Δ*, and *msn2Δ msn4Δ* strains. Although lacZ activity with this reporter was elevated during diauxic growth at 24 h relative to log phase, as expected (48), INAM did not further increase activity (Fig. S5, *A* and *B*, suggesting cAMP levels and Rim15 are not major drivers of the observed CLS extension.

**Figure 7:**
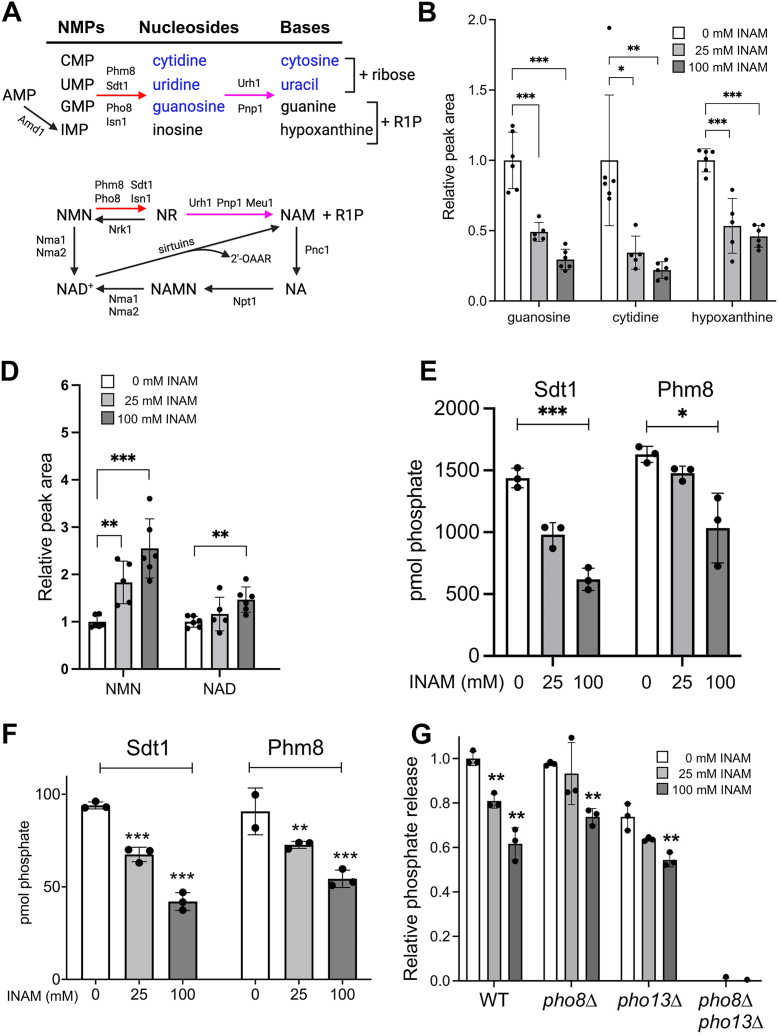
**Inhibition of nucleotidase and alkaline phosphatase activity by INAM**. *A*, nucleoside monophosphate breakdown to bases by nucleotidases (Phm8, Sdt1, and Isn1), alkaline phosphatases (Pho8) to produce nucleosides, followed by the action of purine nucleoside phosphorylase (Pnp1) and uridine nucleosidase (Urh1) to release the bases and either ribose or ribose-1-phosphate. *B*, quantitation of guanosine, cytidine, and hypoxanthine levels following 1 h treatment with 0, 25, or 100 mM INAM. The mean of the control samples without INAM was normalized to 1.0. *C*, nicotinamide mononucleotide (NMN) is broken down to NAM by the same set of enzymes that degrade NMPs as part of the NAD salvage pathway. *D*, quantitation of NMN and NAD ^+^ levels by mass spectrometry following 1 h treatment with 25 or 100 mM INAM. The control was normalized to 1.0. *E*, INAM inhibition of recombinant Sdt1 and Phm8 nucleotidase activity on CMP *in vitro*. *F*, INAM inhibition of recombinant Sdt1 nucleotidase activity on NMN *in vitro*. *G*, INAM inhibition of alkaline phosphatase activity in whole extracts isolated from WT, *pho8Δ*, *pho13Δ*, and *pho8Δ pho13Δ* strains using p-nitrophenyl phosphate (pNPP) as a substrate. In all graphs, error bars represent standard deviations from three biological replicates. Significance *p* values were ∗<0.05, ∗∗<0.005, ∗∗∗<0.005, as calculated using one-way ANOVA. INAM, isonicotinamide; NAM, nicotinamide; NMP, nucleotide monophosphate.

Among the nucleotide-related metabolites significantly reduced by chronic INAM exposure during log phase were cytidine, guanosine, and uridine (Fig. 6*A*), nucleosides primarily produced by dephosphorylation of the ribonucleoside monophosphates, CMP, GMP, and UMP during nucleotide salvage (Fig. 7*A*). Sdt1 and Phm8 are paralogous 5′-nucleotide monophosphate (NMP)-specific nucleotidases (49, 50), and Isn1 is an IMP-specific 5′ nucleotidase (Fig. 7*A*). In addition, the vacuolar alkaline phosphatase Pho8 dephosphorylates 3′-NMPs to generate cytidine, guanosine, and uridine during degradation of RNA by autophagy in response to nitrogen starvation (51). We hypothesized that the depletion of nucleosides caused by INAM was due to inhibition of nucleotidase and/or alkaline phosphatase activity. To test this, BY4741 cultures were grown into log phase, then INAM was added at concentrations of 25 or 100 mM for only 1 h to track acute metabolite changes by mass spectrometry that were likely due to direct enzymatic inhibition (Table S7). Cytidine and guanosine were again significantly reduced by INAM, and in a dose-dependent manner (Fig. 7*B*). Although inosine was not included in the metabolite panel, its downstream base, hypoxanthine, was significantly reduced (Fig. 7*B*). Cytosine and uracil bases were similarly reduced by extended INAM exposure during log phase (Fig. 6*A*), suggesting downstream depletion of the bases, with the surprising exception of guanine.

The same nucleotidases and phosphatases that act on NMPs, Phm8, Sdt1, Isn1, and Pho8, have also been reported to convert NMN to nicotinamide riboside (NR), as depicted in Figure 7*C*; (52, 53)). We found this connection intriguing given the previously observed beneficial effect of INAM on NAD^+^ homeostasis as cells enter the diauxic shift (14). NMN does not cross the yeast cell membrane, so it must first be dephosphorylated into NR and then imported by the NR transporter Nrt1 (54, 55). Preventing NMN dephosphorylation to NR by inhibition of Sdt1, Phm8, Isn1, and Pho8 could potentially “trap” NMN in the cell and shift the flux toward NAD^+^ *via* nicotinamide adenylyltransferases Nma1/Nma1/Pof1 (Fig. 7*C*) (56, 57). We therefore measured changes in NMN and NAD^+^ by mass spectrometry when BY4741 was acutely treated with INAM for 1 h. As shown in Figure 7*D* and Table S7, NMN accumulated in a dose-dependent manner that translated to elevated NAD^+^ at 100 mM INAM, also consistent with our hypothesis of nucleotidase/phosphatase inhibition.

### INAM inhibits nucleotidase and alkaline phosphatase activities

We next tested whether Sdt1 and Phm8 are direct targets of INAM *in vitro* by incubating the recombinant His-tagged enzymes with CMP or NMN substrates and quantifying the release of inorganic phosphate with a colorimetric malachite green phosphate detection assay (58). Sdt1 and Phm8 activity on CMP and NMN was significantly inhibited by INAM concentrations equivalent to those that impacted CLS and nucleoside levels in cultured cells (Fig. 7, *E* and *F*). To evaluate the specificity of nucleotidase inhibition by INAM, we also tested whether Sdt1 was inhibited by NAM at the same concentrations (Fig. S6). Surprisingly, NAM caused significant dose-dependent stimulation of Sdt1 nucleotidase activity with NMN as substrate, demonstrating a clear functional distinction between NAM and INAM, reminiscent of their opposing effects on Sir2 deacetylation activity.

The effect of INAM on alkaline phosphatase activity was next tested by incubating p-nitrophenol phosphate (pNPP) with whole cell extracts from exponentially growing WT, *pho8Δ*, *pho13Δ*, and *pho8Δ pho13Δ* strains. Pho8 and cytosolic Pho13 are the only alkaline phosphatases in *S*. *cerevisiae*, and both utilize pNPP as a substrate *in vitro* (53, 59). Alkaline phosphatase activity was eliminated in the *pho8Δ pho13Δ* extract (Fig. 7*G*), confirming specificity of the assay. INAM moderately weakened alkaline phosphatase activity from the WT and single mutant extracts, suggesting that Pho8 and Pho13 are both weak inhibitory targets. We concluded that upon supplementation, INAM has the potential to inhibit multiple nucleotidase and phosphatase activities that function in nucleotide and NAD^+^ salvage.

**Figure 8:**
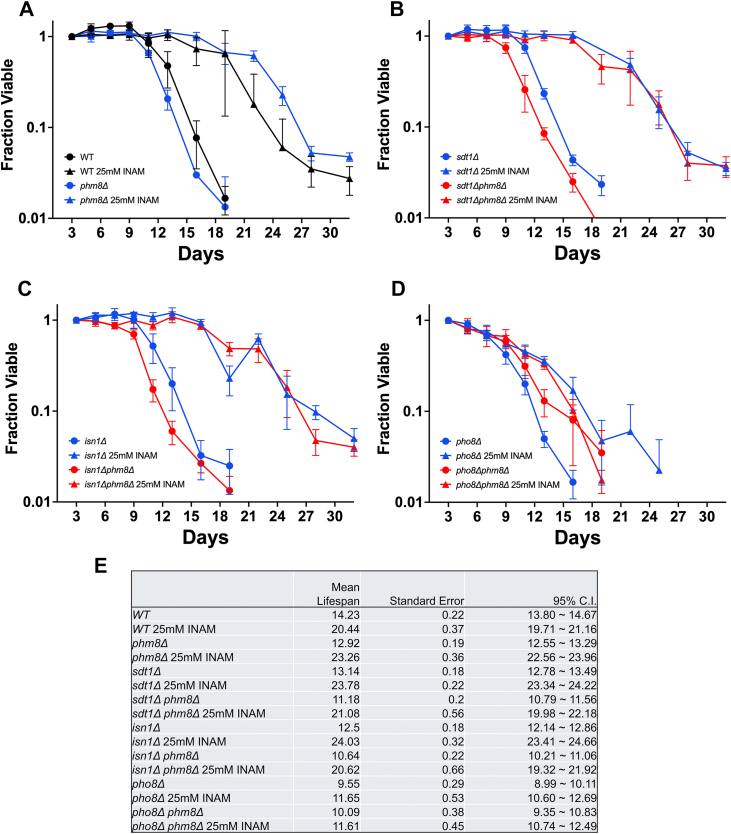
**Pho8 and Phm8 are required for INAM-induced CLS extension**. *A*, CLS assay showing lifespan extension of WT (JS1582) and phm8Δ::natMX4 (SY1478) by 25 mM INAM. *B*, CLS assay showing lifespan extension of *sdt1Δ*:*:kanMX4* (JS1690) and *sdt1Δ*:*:kanMX4 phm8Δ*:*:natMX4* (SY1479) by 25 mM INAM. *C*, CLS assay showing lifespan extension of *isn1Δ*:*:kanMX4* (JS1697) and *isn1Δ*:*:kanMX4 phm8Δ*:*:natMX4* (SY1481) by 25 mM INAM. *D*, CLS assay showing attenuated lifespan extension of *pho8Δ*:*:kanMX4* (JS1694) and lack of lifespan extension of *pho8Δ*:*:kanMX4 phm8Δ*:*:natMX4* (SY1480) by 25 mM INAM. *E*, compilation of mean lifespan, standard error, and 95% confidence intervals for each strain (n = 4 biological replicates). Values were calculated using OASIS 2. CLS, chronological lifespan; INAM, isonicotinamide.

A key prediction was the benefit of INAM on CLS should be lost when its relevant enzymatic targets are absent. Since multiple nucleotidases and phosphatases were inhibited by INAM *in vitro* (Fig. 7), we anticipated there should be significant redundancy *in vivo*. Phm8 is the major 5′NMP nucleotidase utilized for the salvage of ribose during carbon starvation (50). We therefore centered our analysis around this nucleotidase, combining the *phm8Δ* mutation with *isn1Δ*, *pho8Δ*, or *sdt1Δ* to generate a set of double mutant haploid strains for CLS analysis with and without 25 mM INAM supplementation. As shown in Figure 8*A*, CLS of the *phm8Δ* mutant was comparable to WT and fully extended by 25 mM INAM. Similarly, full CLS extension by INAM was observed for *sdt1Δ* (Fig. 8*B*) and *isn1Δ* (Fig. 8*C*), even when combined with *phm8Δ* as double mutants. Although CLS of the *pho8Δ* mutant was reduced relative to WT (Fig. 8, *D* and *E*), INAM still induced significant extension. However, INAM did not extend CLS of the *pho8Δ phm8Δ* double mutant (Fig. 8, *D* and *E*), thus implicating Phm8 and Pho8 as functionally relevant targets that mediate the CLS extension induced by INAM.

## Discussion

### Isonicotinamide and NAD^+^ homeostasis

INAM originally came to our attention because it prevents feedback inhibition of Sir2 activity by NAM, a product of the lysine deacetylation reaction (13). Unexpectedly, INAM also promotes RLS in a Sir2-dependent manner by indirectly maintaining elevated NAD^+^ levels under conditions where NAD^+^ is normally depleted, such as media lacking nicotinic acid (14). In addition, INAM prevents NAD^+^ depletion as cells enter the diauxic shift and stationary phase (14), which led us to test for effects on CLS. INAM at 25 mM strongly extends CLS independently of Sir2 (15), and we now show that it extends lifespan of a quintuple mutant strain lacking all sirtuins (Fig. 2*A*). Although this result precludes Sir2 and other sirtuins mediating the beneficial effect of INAM on CLS, the improved NAD^+^ homeostasis could be contributing to CLS extension independent of sirtuins. Indeed, the NR salvage pathways (Nrk1 and Urh1/Pnp1/Meu1; Fig. 7*C*) were previously reported as critical for maintaining CLS (60). Our new results suggest that INAM inhibition of the nucleotidases Sdt1 and Phm8, or alkaline phosphatases, shifts flux toward intracellular retention of NMN to promote NAD^+^ homeostasis, rather than conversion to NR. The periplasm acid phosphatase, Pho5, converts extracellular NMN to NR but does not significantly affect intracellular NMN dephosphorylation (60).

### Differential effects of INAM and NAM

An earlier screen of the YKO collection for deletion mutants sensitive to nicotinamide (NAM) identified genes that function in sister chromatid cohesion and maintenance of genome stability (61), consistent with the increased mutation frequency we observed with high NAM concentrations (Fig. 2*C*). From the supplemental tables of this earlier paper (61) we extracted 107 NAM-sensitive mutants with normalized colony growth ratios of <0.5 in both screen replicates (61), revealing significantly enriched GO terms of DNA repair (as expected), as well as *de novo* IMP biosynthesis (*ADE1*, *ADE2*, *ADE4*, *ADE6*, and *ADE8*). Genome maintenance genes are not significantly enriched in our screen for INAM-sensitive mutants, consistent with the absence of any mutagenic effect. Interestingly, 21 of the 59 reported NAM-sensitive mutants overlap with our list of INAM-sensitive mutants from Table S4, including *htz1Δ*. Notably, we identify several *ADE* gene mutants as INAM-sensitive, suggesting that NAM and INAM share some level of overlap in their metabolic effects on yeast cells, as might be expected for compounds with such similar chemical structures (Fig. 1*A*). Although NAM at relatively low (1 or 5 mM) concentrations previously shortened CLS (24), most likely due to combined inhibition of Sir2 and Hst1, the extension induced by 25 mM appears to override any inhibitory effect on sirtuins, reminiscent of INAM extending CLS independently of the sirtuins.

Another significant difference between INAM and NAM uncovered from our study is the unexpected stimulation of Sdt1 nucleotidase activity by NAM, rather than the inhibition observed with INAM. The relatively high concentrations needed to observe the inhibition or stimulation of Sdt1 activity suggests a possible low affinity allosteric regulatory binding site for NAM involved in maintaining NAD^+^ homeostasis through a feedback mechanism. INAM, on the other hand, is more likely to interfere with substrate binding. Although there is no published evidence for allosteric regulation of Sdt1 or other yeast nucleotidases, the human cytosolic 5′-nucleotidase II (cN-II), which can utilize NMN as a substrate (62), is known to be allosterically activated by a number of metabolites, including ATP and ADP (63). Further analysis of nucleotidase regulation is therefore warranted. For example, it remains to be determined if Phm8 or Isn1 share the differential NAM/INAM effect on nucleotidase activity using NMN or NMPs as substrates.

### INAM effects on nucleotide metabolism

One of the key clues that INAM modifies nucleotide metabolism is the discovery that multiple INAM-sensitive elongation factor mutants also display sensitivity to MPA or 6-AU, compounds known to decrease guanine nucleotide pools by inhibiting IMPDH (40, 64). Previous research has shown that reduced elongation activity in these mutants, along with lower nucleotide pools, leads to synthetic growth defects. Based on these findings, we hypothesized that INAM suppresses growth of elongation factor mutants by further compromising nucleotide pools. This idea is supported by additional genetic findings: INAM-sensitive mutants are found in serine, threonine, and aspartate synthesis, as well as the transsulfuration and *de novo* purine biosynthesis (ADE) pathways, all of which connect with nucleotide synthesis *via* the 1C metabolism pathway. Accordingly, we quantified nucleotide and precursor levels in cells continuously exposed to 25 mM INAM across a time course covering the transition into stationary phase, a critical period for chronological longevity determination. We observed significant reductions in nucleosides and bases during log phase (Fig. 6*A*), followed by pronounced drops in dNTPs at 24 h and then both NTPs and dNTPs at 96 h. Brief INAM exposure of log phase cells for 1 h also led to marked depletion of guanosine and cytidine, implicating nucleotidases such as Sdt1, Isn1, and Phm8, as well as the alkaline phosphatase Pho8, as likely INAM targets.

Notably, INAM failed to extend CLS of a *phm8Δ pho8Δ* double mutant (Fig. 8), suggesting these two enzymes are functional targets *in vivo*. However, since not every mutant combination among the four candidate enzymes was tested, additional combinations that block INAM-induced CLS extension are possible. Pho8 could be the most central target, based on the partial CLS extension of the *pho8Δ* mutant, and combining *pho8Δ* with any one of the nucleotidase gene deletions may be sufficient to block the INAM effect. Sdt1 is a top candidate given that it is also inhibited by INAM *in vitro* and is paralogous with Phm8. Alternatively, it is possible that the *phm8Δ pho8Δ* double mutant is uniquely refractory to INAM because CLS extension requires the simultaneous salvage of base and ribose moieties derived from 5′-NMPs *via* Phm8 and 3′NMPs *via* Pho8. Finally, we cannot rule out the possibility that INAM alters the activity of additional enzymatic steps of *de novo* or salvage pathways for nucleotide metabolism, or any enzyme that interacts with NAD(H) or NADP(H), especially with the structural similarity between INAM and NAM.

### Nucleotide pool effects on lifespan

Given the numerous transcription elongation mutants identified as INAM-sensitive, we initially hypothesized that the elongation defects were contributing to the CLS extension. In multicellular organisms, the rate of transcriptional elongation increases with age and genetic manipulations that slow elongation tend to extend lifespan (65). It remains unclear if alterations in elongation have any impact on yeast CLS. In our study, deletion of *SWR1* or *HTZ1* was not sufficient to extend CLS and did not block the CLS extension induced by 25 mM INAM, even though the *htz1Δ* mutant growth was slowed at this concentration. Therefore, any combined impacts on transcriptional elongation by these mutants and/or INAM were likely not driving the lifespan extension.

Alternatively, perturbation of nucleotide pools by INAM during the post log phase may induce mild DNA replication stress. This phenomenon is reminiscent of guanine nucleotide depletion in T and B lymphocytes, which leads to S-phase arrest, replication stress, and apoptosis due to replication fork stalling (66, 67). Similarly, hydroxyurea (HU) induces replication stress by inhibiting ribonucleotide reductase and consequently depleting the dNTP pool. Notably, HU has been shown to extend CLS through a mechanism independent of classical stress resistance pathways, instead promoting entry into quiescence (68). The resulting replication stress is proposed to increase the proportion of cells that transiently arrest their cell cycle during growth, thereby enhancing the probability of cell cycle exit and entry into quiescence (68). In addition, depletion of guanylic nucleotides with MPA has been shown to cause a mother-daughter cell separation defect and the emergence of bi-budded cells, which are observed in cells entering quiescence (69). Thus, both HU and MPA appear to facilitate entry into quiescence as cultures approach stationary phase. Consistent with these observations, INAM-induced replication stress may promote CLS by enhancing entry into quiescence.

### Isonicotinamide implications for human health

Could INAM or its derivatives be used as an aging intervention in humans? INAM is commonly used as a backbone for the synthesis of various drugs, as well as cocrystallization with existing compounds to make them more bioavailable (70, 71). Examples include the MEK1/2 inhibitor pimasertib (72), xanthine oxidase inhibitors where the INAM moiety is the key functional region (73), and new GSK-3 inhibitors in development as Alzheimer’s treatments (74). INAM itself is also known to inhibit the activity of poly(ADP-ribose) polymerase 1 in mammalian cells, similar to the effect of NAM (75). Indeed, the strong elevation of NAD^+^ observed in lung cancer cells upon INAM treatment is likely caused by poly(ADP-ribose) polymerase 1 inhibition, though nucleotidases/phosphatase inhibition could also be involved, as cN-II and cN-III are both capable of utilizing NMN and nicotinic acid mononucleotide (NaMN) as substrates (62). Given its chemical similarity to NAM, INAM also has the potential to target other enzymes that interact with the NAD^+^ family of metabolites. As INAM-derived therapeutics are used more in clinical trials, it will therefore be of interest to track the impacts on NAD^+^ levels and nucleotide pools, as well as health span parameters.

## Experimental procedures

### Yeast strains and media

The primary “wild-type” laboratory strain in this study was BY4741 (*MAT*a *his3Δ1 leu2Δ0 met15Δ0 ura3Δ0*) (76), and most gene deletion mutants were obtained from the isogenic YKO collection (77). JS1582 is a *MET15*^+^ version of BY4741. The *phm8Δ* strain, SY1478, was created by replacing *PHM8* with *natMX4* using the PCR-mediated knockout approach by transformation. Similarly, *PHM8* was deleted and replaced with *natMX4* in *sdt1Δ*:*:kanMX4*, *pho8Δ*:*:kanMX4*, or *isn1Δ*:*:kanMX4* versions of SY1478 to generate double mutants. The *ade2-101* mutation in YPH499 and YCB498 was repaired by transformation with an *ADE2* PCR product spanning the ochre mutation and selecting for Ade^+^ colonies to yield SY1043 and SY1044, respectively. The reversion was confirmed by Sanger DNA sequencing. The lacZ reporter strains CDV120, CDV121, and CDV123 were kindly provided by Claudio De Virgilio. Detailed strain information is provided in Table S4. SC media with 2% glucose as a carbon source was used for most assays, with yeast extract peptone dextrose (YPD) used for cell propagation and selection prior to the experiments. All liquid cultures and agar plates were grown at 30 °C.

### CLS assays

Ten milliliters of SC media with 2% glucose and relevant supplements was inoculated with 100 μl of overnight culture. Liquid cultures were incubated on a rotating drum (TC-7, New Brunswick Scientific) in glass tubes with metal caps allowing for gas exchange. After 72 h, the first measurement of colony forming units on YPD agar plates was made and this was treated as day 3 for the experiment (100% starting viability), to which all the other data was normalized. Measurements were taken every 3 to 4 days as previously described (30, 78). At each time point, 20 μl of the cell suspension was removed from each tube and 10-fold serially diluted three times with sterile water. Next, 2.5 μl of each dilution (1:10, 1:100, 1:1000) was spotted onto a YPD plate. After 16 h, images of the spots were taken under a Nikon Eclipse E400 brightfield microscope at 30x magnification. Microcolonies within the spots were automatically counted from the digital images using OrganoSeg (79), with the parameters adjusted for yeast colony counting (30). After accounting for the dilution factor, colony numbers from each day were divided by the number of colonies from the first time point (day 3) to give the viability metrics.

### SGA screen

The *MAT*a haploid YKO collection, consisting of 4648 strains organized into 18 plates in 384-well format, was spotted onto Nunc Omnitray single-well plates containing YPD-agar with 200 μg/ml G418 using a floating pin manual replicator (VP 384FS, V&P Scientific). After 3 days growth, the replicator was used to transfer cells from colonies on the YPD plates to SC-agar Omnitrays supplemented with 0, 25, 50, 75, or 125 mM INAM. Colony growth for the 0, 25, 50, and 75 mM INAM plates was imaged with a Fluor Chem Q gel documentation system (ProteinSimple) after 2 days, while the 125 mM plates were imaged after 3 days incubation. The screen was performed in duplicate. SGAtools (http://sgatools.ccbr.utoronto.ca/) was then used to quantify colony growth from jpeg images, comparing the INAM-containing plates to control plates without INAM (28). The score value of −0.3 was used as a cutoff for INAM sensitivity, and to limit false positives we required that both replicates reach this threshold for at least the 75- and 125-mM conditions. Most mutants with deletions of dubious open reading frames and genes without a confirmed function were omitted in further analysis, as their sensitivity would not provide any valuable information about the mechanism of action of INAM.

### Growth assays

For qualitative spots test growth assays, strains were patched onto YPD agar plates and grown overnight. The cell patches were resuspended in water at an *A*_600_ of 1.0, then 10-fold serially diluted. Next, 2.5 μl of each dilution was spotted onto SC agar plates containing 0, 25, 50, 75, or 125 mM INAM, and photos taken after 2 days of growth.

For ED_50_ assays, 10 ml SC cultures were incubated overnight in the roller drum, then added to wells of a 96-well plate at a starting *A*_600_ of 0.1 in SC media containing a range of INAM concentrations (0–240 mM; 2-fold dilutions) and other supplements as indicated. The initial *A*_600_ was measured with a Spectra Max M2 plate reader (Molecular Devices). Plates were then sealed with a sterile, gas-permeable membrane and incubated in a heated microplate shaker (Southwest Science, SBT1500-H) at 30 °C for 8 h at 900 RPM. Final *A*_600_ was measured and the initial *A*_600_ subtracted. Values were then normalized to fit the range 0 to 100%, where the control condition (no INAM) was treated as 100% and the highest INAM concentration as 0. These normalized growth values were then plotted on a graph using Origin 2018 (OriginLab) and the sigmoidal fit was used to model a dose-response curve fitting the obtained data. Effective dose (ED_50_) was calculated as the concentration at which there was 50% viability. The 95% confidence intervals were also calculated by the Origin software from the 95% confidence bands. An additional benefit of this ED_50_ approach is precisely determining if mutant strains with intrinsically slow growth are sensitive to INAM, due to the assay being internally normalized to growth of a given strain in the control condition (no INAM), thus allowing direct comparison of strains with different growth rates.

Synergy assessment was performed similarly to the IC_50_ assays, with a combination of increasing MPA and INAM concentrations added in a matrix to a 96-well plate. Methanol vehicle concentration was adjusted to be the same at all MPA concentrations used. SynergyFinder (80) was used to analyze the synergy between compounds using baseline correction and a zero interaction potency (ZIP) model of synergy determination (81).

### Measurement of metabolite abundance by mass spectrometry

Cells were grown in 10 ml SC 2% glucose with or without 25 mM INAM on a roller drum. Cultures were started from overnight liquid cultures at *A*_600_ adjusted to 0.05. Cell densities of cultures were measured before collection for normalization of metabolites. Cells were collected at log phase (∼5 h), 24 h, and 96 h. Alternatively, log phase cultures were supplemented with 0, 25, or 100 mM INAM for 1 h, followed by immediate cell harvest. Intracellular metabolites were isolated as previously described (82), with the extraction protocol modified for yeast. From these cultures, 9 ml was added to 20 ml of 100% cold (−80 °C) LC/MS-grade methanol kept on dry ice (final methanol concentration: ∼70% v/v). Samples were then kept on dry ice unless indicated otherwise. The quenched cell suspensions were spun down at 2000*g* in an Eppendorf 5810R centrifuge cooled to −9 °C for 5 min. The cell pellets were resuspended in 1 ml 80% methanol (LC/MS grade) and transferred to screw-top microfuge tubes containing 0.5 mm acid-washed glass beads. A Mini-Beadbeater (Biospec products) was used to vigorously shake the microfuge tubes at 4 °C for three cycles of 45 s with 30-s pauses. Each tube was then punctured at the bottom with a needle, placed into a glass tube and spun down at −9 °C for 5 min at 2000 RPM in the Eppendorf 5810R centrifuge to collect cell extracts. Beads were washed twice with 1.5 ml 80% methanol and spun down the same way (4 ml total pooled volume). The pellet was resuspended by vortexing and kept on ice for 15 min, followed by centrifugation at 3100*g*. Supernatants were transferred to two 2-ml microfuge tubes, and evaporated in a Vacufuge Concentrator 5301 (Eppendorf, Hamburg, Germany) at 30 °C until 20% volume remained. Remaining water was then removed by lyophilization.

Metabolites were analyzed as described previously (83, 84), with a selected reaction monitoring liquid chromatography-tandem mass spectrometry (LC-MS/MS) method with positive/negative ion polarity switching using a Xevo TQ-S mass spectrometer. MassLynx 4.1 software was used to calculate the peak areas for each metabolite. Peak areas were normalized to respective *A*_600_ of each sample. These normalized peaks were subsequently scaled relative to the mean peak area in the control condition at each time point (Tables S6 and S7).

### Enzymatic assays

Alkaline phosphatase activity was measured from whole cell extracts as previously described (53). WT (BY4741), *pho8Δ* (SY1124), *pho13Δ* (SY1136), and *pho8Δ pho13Δ* (SY1137) strains were grown overnight in 10 ml YPD to an *A*_600_ of 2 to 3. Cell pellets were washed (0.85% NaCl, 1 mM PMSF) and then disrupted in 1.8 ml lysis buffer (20 mM Pipes, 0.5% Triton X-100, 50 mM KCl, 100 mM potassium acetate, 10 mM MgSO_4_, and 10 μM ZnSO_4_ with 1 mM PMSF added just before use) using a Biospec Mini-Beadbeater. Following centrifugation, 200 μl of supernatant was added to 300 μl of reaction buffer (333 mM Tris–HCl, pH 8.8, 133 mM MgSO_4_, 13.3 μM ZnSO_4_, 0.53% Triton X-100, with or without 1.66 mM p-nitrophenyl phosphate) in a microfuge tube and incubated for 15 min at 37 °C. Next, 500 μl of stop buffer (1M Glycine, 1M KOH, pH 11) was added to the reaction tubes, which were centrifuged again for 5 min at 4 °C. Two hundred microliters of each reaction was then transferred into a 96-well plate and read at *A*_*4*00_ on a Molecular Devices Spectramax M5 plate reader. Activity was normalized to the *A*_600_ of each culture.

Nucleotidase activity of recombinant Sdt1 and Phm8 was measured by quantifying the release of phosphate from *in vitro* reactions. C-terminally His-tagged Sdt1 (E−20SDT1) was obtained from Echelon Biosciences Inc. Phm8 was cloned into the *Nde*I site of pET-16b plasmid and expressed in BL21(DE3) *Escherichia coli* as a C-terminal 10xHis-tagged protein. The recombinant protein was purified using Ni-NTA agarose beads and the pooled fractions were dialyzed with 50 mM Tris–HCl, pH 7.5, 50 mM NaCl, 2 mM β-mercaptoethanol, 50% glycerol in 30 kDa cutoff dialysis tubing. The Sigma-Aldrich Phosphate Assay Kit (MAK308-1KT) was used to determine nucleotidase activity. Subsequently, 0.5 μg Sdt1 or 0.5 μg Phm8 was incubated with 100 μM CMP (Tokyo Chemical Industry, YK6JM-MI) or NMN (Cayman, 16411) for 30 min at 37 °C in 50 μl of reaction buffer (50 mM Tris–HCl, pH 7.5, 5 mM MgCl_2_) in a 96-well plate. CMP and NMN were left out as negative controls. Reactions were then incubated with 100 μl of malachite green solution (Sigma-Aldrich, MAK308A-KC) for 20 minutes at room temperature (58). Absorbance was then measured at 620 nm using the Spectramax M5 plate reader. Dilutions of a 1 mM phosphate standard were used as a standard curve for quantitation of pmol phosphate released.

## Data availability

All data supporting the findings of this study are available within the article and its supplementary information files.

## Supporting information

This article contains supporting information.

## Conflict of interest

The authors declare that they have no conflicts of interest with the contents of this article.
