# Supplementary material for: Chronological lifespan extension and nucleotide salvage inhibition in yeast by isonicotinamide supplementation
Source: J Biol Chem. 2026 Jan 13;302(3):111158. doi: 10.1016/j.jbc.2026.111158 (PMC12876586; doi:10.1016/j.jbc.2026.111158)

Supporting Information

Chronological lifespan extension and nucleotide salvage inhibition in yeast by isonicotinamide supplementation

Agata Kalita, Christopher Letai, Elisa Enriquez Hesles, Lindsey N. Power, Swarup Mishra, Shekhar Saha, Manikarna Dinda, Dezhen Wang, Pankaj K. Singh, and Jeffrey S. Smith

Figures S1 – S6

Excel files:

Table S1. List of candidate genes from the INAM sensitivity screen at concentrations of 25, 50, or 75 mM.

Table S2. List of candidate genes from the INAM sensitivity screen at 125 mM.

Table S3. List of significantly enriched GO terms of the genes isolated from the 25, 50, and 75 mM INAM sensitivity screens.

Table S5. OASIS 2 generated statistical analysis for each CLS experiment found in the figures for this study.

Table S6. Mass spectrometry results for chronic INAM treatment. Normalized peak values for each growth condition. Peak areas were normalized to respective OD_{600} of each sample. These normalized peaks were subsequently scaled relative to the mean peak area in the control condition at each time point.

Table S7. Mass spectrometry results for acute (1hr) INAM treatment. Normalized peak values for each growth condition. Peak areas were normalized to respective OD_{600} of each sample. These normalized peaks were subsequently scaled relative to the mean peak area in the control condition at each time point.

Word file:

Table S4. List of yeast strains used in the study.

Fig. S1. Chemical genetic screen for INAM sensitive gene deletion mutants from the yeast knockout (YKO) strain collection. **A)** The haploid yeast knockout collection was pinned onto control SC plates or SC plates with increasing concentrations of INAM using a manual pinning tool. **B)** Zoomed in portion of plate 7 showing INAM sensitivity of the *tho2*Δ strain (yellow arrow). Tho2 is a transcription elongation factor. **C)** Scatter plot of scores from each deletion strain obtained in experiment 1 (x-axis) and experiment 2 (y-axis). Strains with SGAtools fitness scores lower than -0.3 in both replicates were considered sensitive to a given INAM concentration (representative graph for 75 mM INAM). **D)** Venn diagram showing the overlap of strains scored as sensitive between different INAM concentrations.

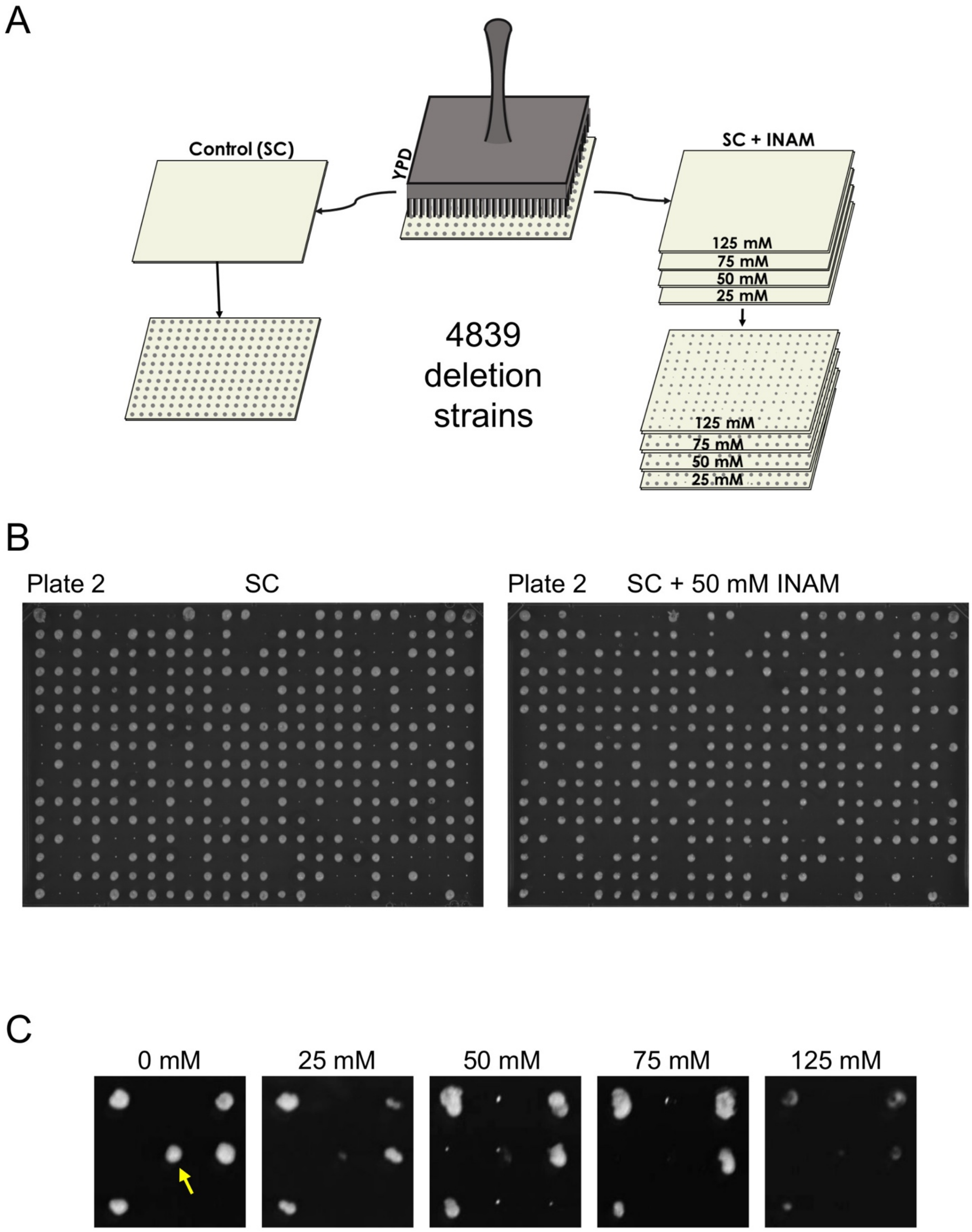
A
Control (SC)
YPD
SC + INAM
125 mM
75 mM
50 mM
25 mM
4839
deletion
strains
B
Plate 2
SC
Plate 2
SC + 50 mM INAM
C
0 mM
25 mM
50 mM
75 mM
125 mM

Fig. S2. Confirmation of candidate mutants from the INAM sensitivity screen using spot test growth assays. The normalized growth scores from SGAtools are indicated for the two independent screens (performed sequentially). Scores for 25, 50, and 75 mM plates (shown) were from day 2 images, while fitness scores for 125 mM plates were from day 3 images. Fitness scores below the -0.3 cutoff are shaded in green. White lines separate individual strains, indicating they were not necessarily grown next to each other on the same plate. The BY4741 control dilution series in this figure is shared with Figure S3B. Similarly, the BY4741 control at the bottom of S3A is shared with the bottom of S4.

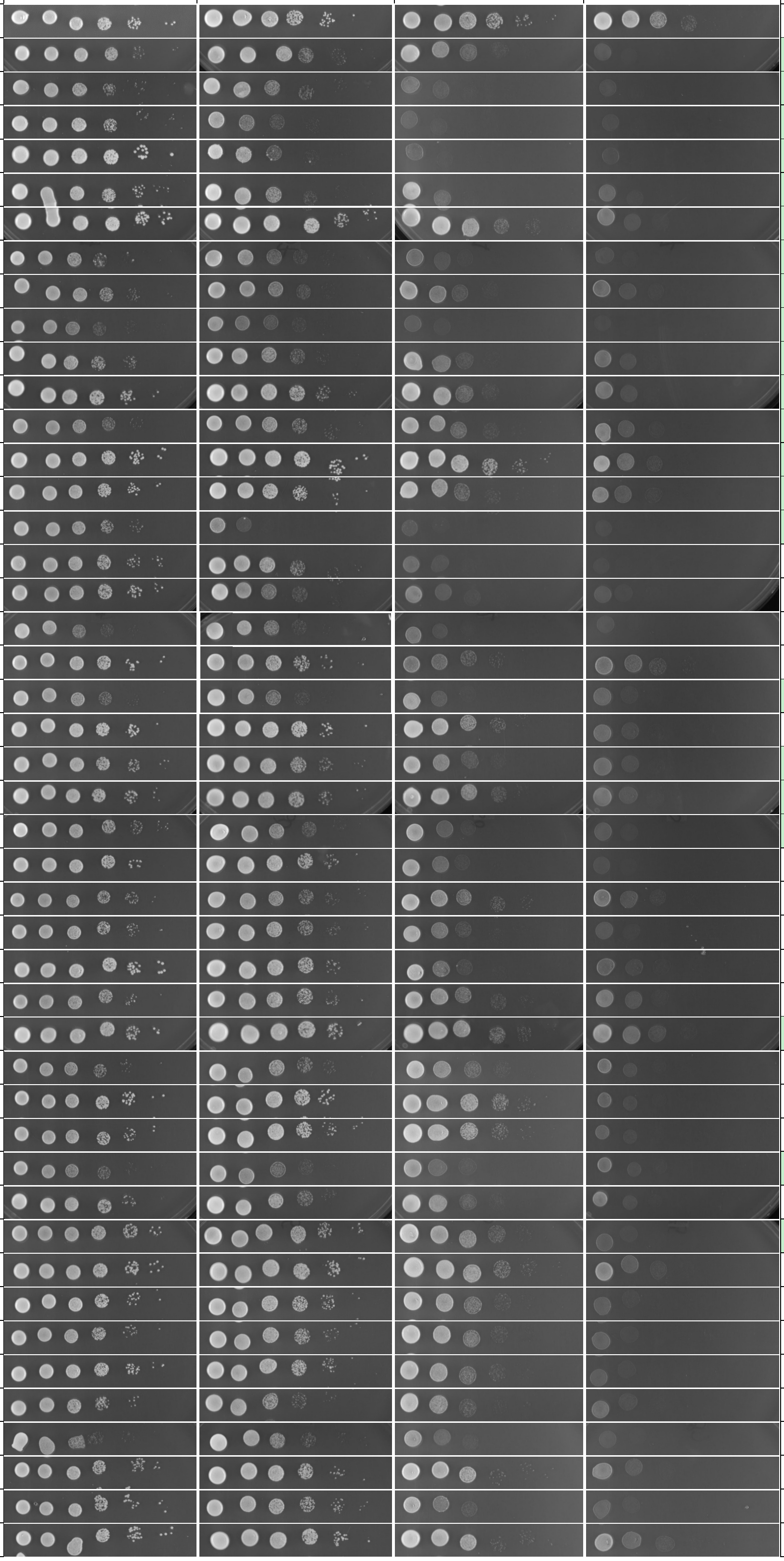

ORFΔ	GeneΔ	SC	SC + 25 mM INAM	SC + 50 mM INAM	SC + 75 mM INAM	Experiment #1				Experiment #2			
						Day 2			Day 3	Day 2			Day 3
						25 mM	50 mM	75 mM	125 mM	25 mM	50 mM	75 mM	125 mM
BY4741													
YOL051W	GAL11					-0.351	-0.443	-0.705	-0.705	-0.725	-0.725	-0.725	-0.758
YPL129W	TAF14					-0.345	-0.886	-0.845	-0.897	-0.669	-0.914	-1.144	-1.127
YNL139C	THO2					-0.637	-1.292	-1.734	-1.735	-1.909	-1.962	-1.962	-1.479
YBL094C						-1.031	-1.031	-1.031	-1.031	-0.754	-0.968	-0.968	-0.939
YDL117W	CYK3					-0.445	-0.502	-0.696	-0.454	-1.349	-1.648	-1.648	-0.655
YDR011W	SNQ2					-0.572	-0.474	-1.445	-1.445	-0.759	-0.607	-1.441	-1.476
YDR017C	KCS1					-1.017	-1.017	-1.017	-1.017	-0.739	-1.096	-1.402	-1.341
YDR226W	ADK1					-0.811	-0.855	-0.739	-0.701	-0.541	-0.608	-0.730	-0.314
YGL095C	VPS45					-1.930	-1.930	-1.930	-1.925	-0.760	-1.413	-1.413	-0.913
YKL139W	CTK1					-0.546	-0.592	-0.585	-0.501	-0.456	-0.480	-0.435	-0.658
YNL079C	TPM1					-0.611	-0.801	-1.224	-1.093	-1.045	-1.097	-1.314	-1.13
YKR029C	SET3					-0.308	-0.322	-0.438	-0.512	-0.332	-0.332	-0.332	-0.344
YER111C	SWI4					-0.889	-0.945	-1.323	-1.323	-1.227	-1.328	-1.962	-1.777
YER139C	RTR1					-0.592	-0.592	-0.592	-0.592	-0.939	-1.069	-1.069	-0.946
YLR399C	BDF1					-0.819	-0.819	-0.819	-0.807	-0.634	-0.634	-0.634	-0.468
YOL012C	HTZ1					-0.036	-0.734	-0.741	-0.742	-0.815	-0.815	-0.815	-0.833
YHL031C	GOS1					-0.218	-0.677	-0.714	-0.804	-0.360	-0.976	-1.209	-1.032
YEL044W	IES6					-0.125	-1.159	-1.384	-1.442	-0.061	-0.554	-0.742	-0.731
YER052C	HOM3					-0.099	-0.616	-0.989	-0.99	-0.105	-0.387	-0.443	-0.379
YPL106C	SSE1					-0.668	-1.284	-1.013	-1.292	-0.190	-0.891	-1.313	-0.504
YBR231C	SWC5					-0.219	-0.512	-0.948	-1.114	-1.391	-1.304	-1.391	-1.388
YDR245W	MNN10					-0.302	-0.555	-1.235	-1.331	0.104	-0.312	-0.890	-1.189
YDR392W	SPT3					-0.958	-0.688	-0.969	-1.128	-0.180	-0.429	-0.676	-0.968
YGR063C	SPT4					-0.599	-1.079	-1.079	-1.052	-0.083	-0.311	-0.653	-1.177
YNL107W	YAF9					-0.162	-0.632	-0.632	-0.633	-0.209	-0.445	-0.445	-0.447
YCR094W	CDC50					-0.145	-0.482	-0.595	-1.207	-1.044	-0.977	-1.053	-1.062
YHL025W	SNF6					-0.006	-0.661	-0.766	-0.766	-0.503	-0.924	-0.924	-0.891
YER155C	BEM2					-0.110	-0.752	-0.840	-0.95	-1.016	-1.016	-1.016	-1.009
YLR337C	VRP1					0.023	-1.073	-1.441	-1.442	-0.052	-1.031	-1.443	-1.439
YLL012W	YEH1					-0.488	-0.001	-0.771	-1.905	-0.090	-0.447	-1.157	-1.535
YIL128W	MET18					-0.166	-0.124	-0.331	-0.605	-0.107	-0.571	-0.927	-0.93
YIL097W	FYV10					-0.023	0.013	-0.978	-0.95	-0.045	-0.229	-1.414	-1.422
YLR182W	SWI6					-0.235	-0.348	-0.564	-0.565	-0.068	-0.236	-0.753	-0.545
YLR226W	BUR2					-0.330	-0.194	-0.478	-1.01	-0.374	-0.816	-1.065	-0.735
YBL027W	RPL19b					-0.280	-0.227	-0.676	-0.857	-0.162	-0.132	-0.590	-0.741
YBR077C	SLM4					-0.320	-0.168	-0.448	-0.353	-0.068	-0.483	-1.005	-1.045
YDR293C	SSD1					0.493	0.067	-0.666	-1.256	-0.557	-0.761	-1.546	-1.961
YDR334W	SWR1					0.542	0.270	-0.551	-0.93	-0.896	-1.155	-1.616	-1.591
YGL025C	PGD1					-0.246	-0.061	-0.683	-0.738	0.012	-0.127	-0.329	-1.017
YDR485C	SWC2					0.210	-0.050	-0.471	-0.6	-0.107	-0.190	-0.611	-0.649
YAL047C	SPC72					-0.144	-0.575	-1.038	-1.962	6.561	6.701	-3.664	-1.973
YNL059C	ARP5					0.642	-0.151	-0.629	-1.214	0.270	-0.938	-1.420	-1.493
YNL147W	LSM7					0.003	-0.162	-0.501	-0.86	-0.434	-0.527	-0.734	-0.985
YMR091C	NPL6					0.260	-0.143	-0.537	-0.552	-0.740	-0.817	-0.892	-0.737
YOL114C	PTH4					0.235	-0.110	-0.739	-1.164	-0.350	-0.473	-1.155	-1.323

Fig. S3. Confirmation of additional candidate mutants from INAM sensitivity screen. **A)** Mutants that confirmed as INAM sensitive. Each experiment has its own BY4741 WT control. **B)** Mutants that did not confirm as INAM sensitive. SGAtools fitness scores for the two screens are indicated to the right, as in Fig. S2.

A

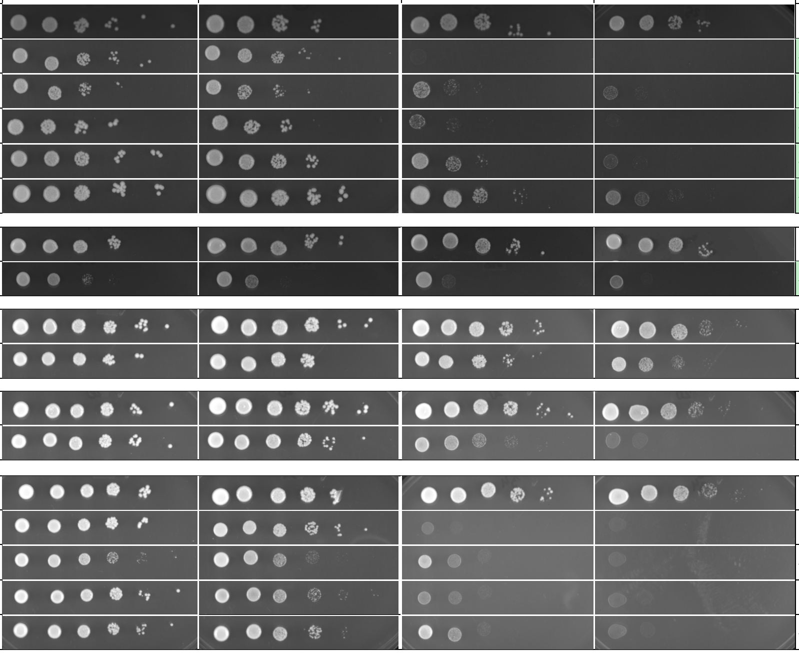

ORFΔ	GeneΔ	SC	SC + 25 mM INAM	SC + 50 mM INAM	SC + 75 mM INAM	Experiment #1				Experiment #2			
						Day 2			Day 3	Day 2			Day 3
						25 mM	50 mM	75 mM	125 mM	25 mM	50 mM	75 mM	125 mM
BY4741													
YDR364C	CDC40					-0.999	-0.999	-0.999	-0.996	-0.329	-0.538	-0.480	-0.521
YDR532C	KRE28					-0.481	-0.307	-0.611	-0.915	-1.454	-1.454	-1.454	-0.87
YCR020W-B	HTL1					-0.473	-0.473	-0.436	-0.599	-0.643	-0.643	-0.643	-0.629
YDR138W	HPR1					-0.555	-0.640	-0.897	-0.85	-0.029	-0.401	-0.774	-0.744
YMR269W	TMA23					-0.517	-0.156	-0.330	-0.943	-0.168	-0.507	-0.763	-0.967
BY4741													
YJL176C	SWI3					-0.754	-0.548	-0.566	-0.672	-0.049	-0.957	-0.957	-0.908
BY4741													
YCR002C	CDC10					0.030	-0.341	-0.518	-0.982	-0.060	-0.465	-0.946	-1.119
BY4741													
YLR025W	SNF7					0.226	-0.298	-1.096	-0.563	-0.833	-0.959	-1.187	-0.947
BY4741													
YML112W	CTK3					0.512	0.191	-0.565	-1.112	0.107	-0.493	-1.794	-1.244
YHL011C	PRS3					-0.175	-0.084	-0.727	-1.404	2.162	-1.469	-2.340	-0.986
YGR155W	CYS4					0.865	0.521	-0.349	-1.096	-5.046	-5.046	-5.046	-1.973
YHR041C	SRB2					-0.001	-0.262	-0.943	-1.535	2.669	6.669	-5.046	-1.973

B

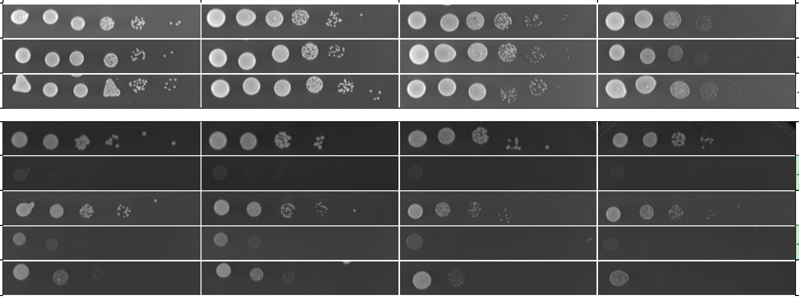

ORFΔ	GeneΔ	SC	SC + 25 mM INAM	SC + 50 mM INAM	SC + 75 mM INAM	Experiment #1				Experiment #2			
						Day 2			Day 3	Day 2			Day 3
						25 mM	50 mM	75 mM	125 mM	25 mM	50 mM	75 mM	125 mM
BY4741													
YBL103C	RTG3					-0.023	0.351	-0.610	-0.489	0.149	0.008	-0.424	-0.817
YOL115W	PAP2					-0.159	-0.182	-0.306	-0.306	-0.392	-0.442	-0.561	-0.822
BY4741													
YEL046C	GLY1					-0.926	-1.071	-0.719	-0.488	-0.852	-0.852	-0.852	-0.327
	GLY1 Day 7												
YBR114W	RAD16					-0.645	-0.645	-0.645	-0.502	-0.532	-0.532	-0.430	-0.317
	RAD16 Day 7												

Fig. S4. Strains not identified by the screen but confirmed as sensitive in the spot test growth assays. These additional strains were chosen based on identity of confirmed strains from the screen and the literature.

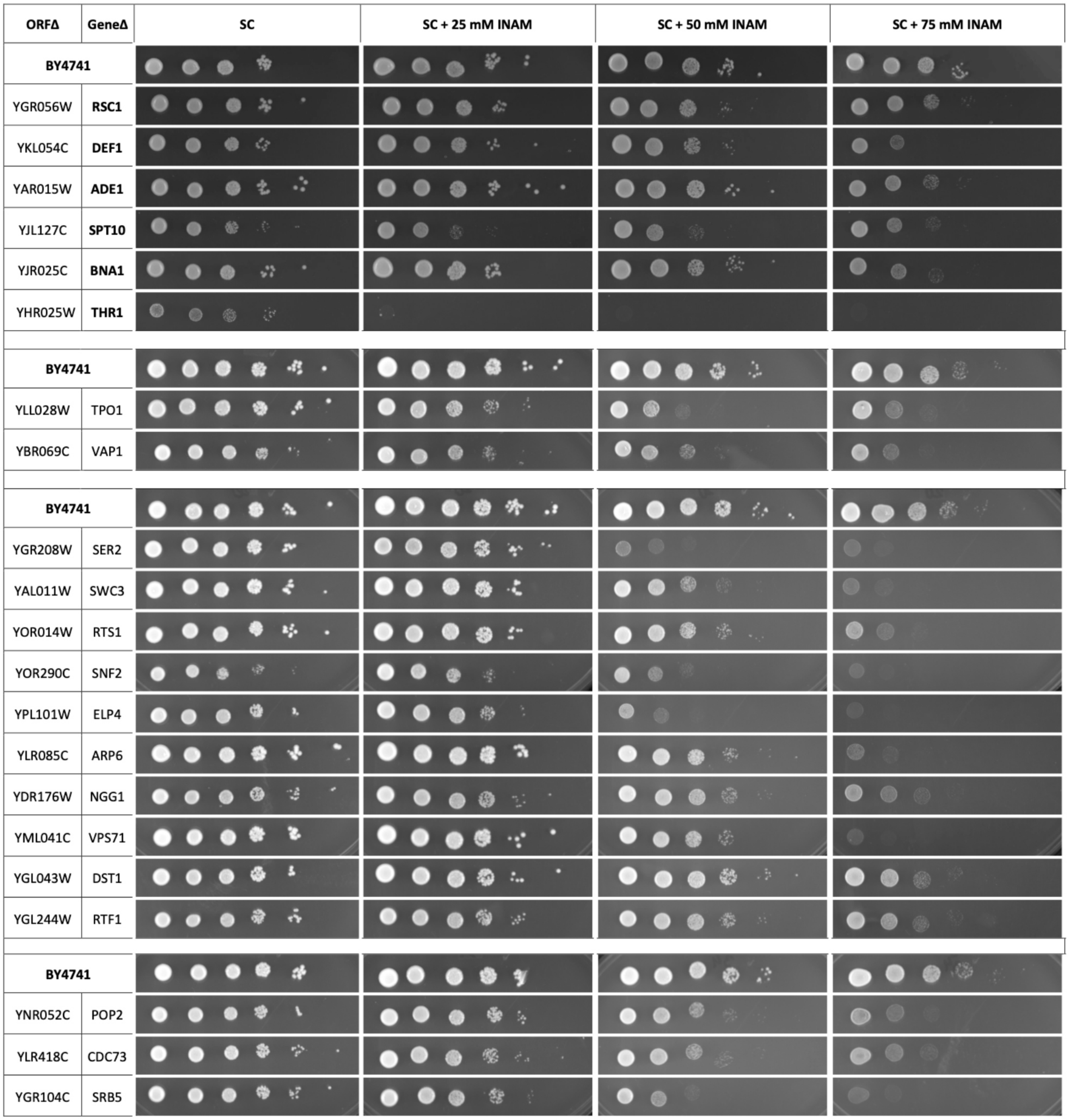

Fig. S5. Isonicotinamide does not activate transcription of a lacZ reporter gene regulated by a STress Response Element (STRE) motif in the promoter. **A)** β-galactosidase activity levels from whole yeast cell extracts of WT, *rim15*Δ, and *msn2*Δ *msn4*Δ mutants incubated with 25 mM INAM for 6 hours in log phase. **B)** β-galactosidase activity levels from whole yeast cell extracts of WT, *rim15*Δ, and *msn2*Δ *msn4*Δ mutants incubated with 25 mM INAM for 24 hours grown into late diauxic shift. No significant differences were observed for each strain following INAM supplementation (one-way ANOVA) except for a slight decrease for the WT strain at 24 hr.

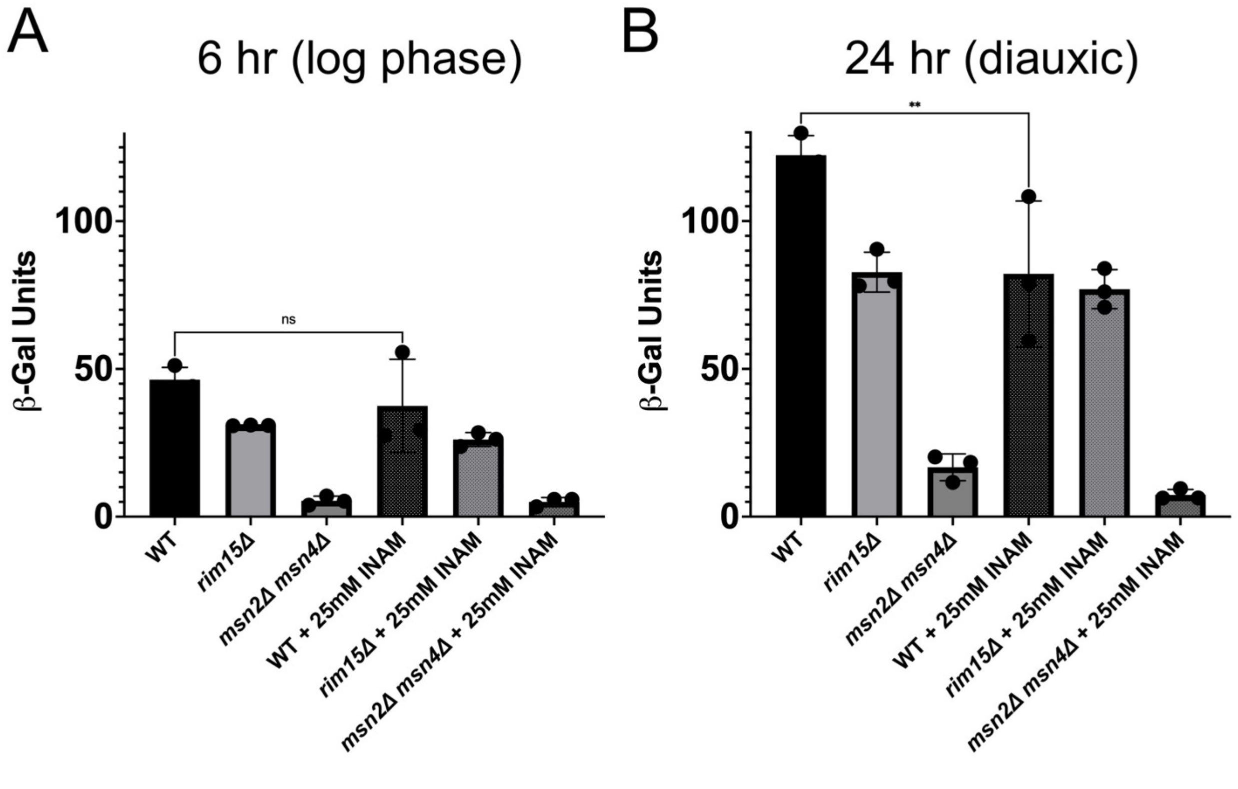

Fig. S6. Stimulation of Sdt1 nucleotidase activity by INAM using NMN as a substrate. Recombinant Sdt1 was treated with NAM at 25mM and 100mM concentrations. Activity is represented as phosphate released (pmol) during the 30 min reaction. Inhibition of activity by INAM is shown as a control for specificity. White bars indicate no treatment. Light gray bars indicate 25 mM, and dark gray bars indicate 100 mM. ****p-values <0.0001, as calculated using one-way ANOVA.

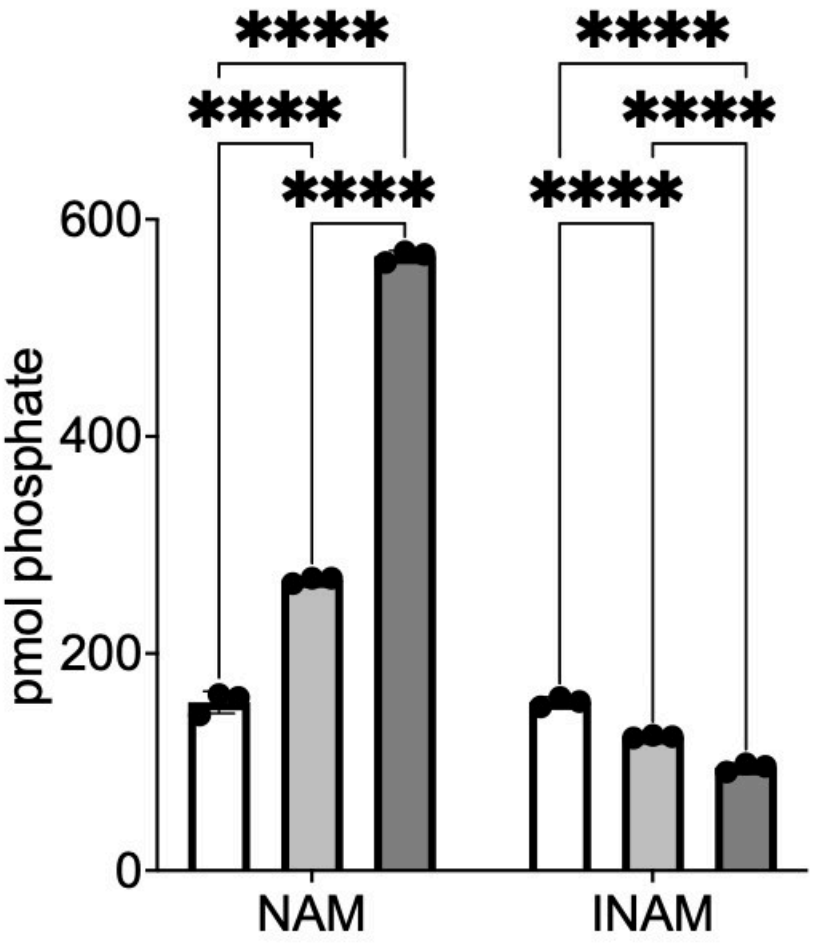

Supplement: Supplementary Material 1 [file mmc1.pdf]
